# Antimicrobial action of ozonated water and photodynamic therapy with sonic activation in root canals infected with *Enterococcus faecalis*

**DOI:** 10.4317/jced.57909

**Published:** 2022-06-01

**Authors:** Isabel-Alessandra-Miranda Nunes, Tânia-Maria-Gaspar Novais, Patrick-Pereira Garcia, Wallison-Sousa Silva, Rudys-Rodolfo-de Jesus Tavarez, Claudia-de Castro Rizzi, Ceci-Nunes Carvalho, Etevaldo-Matos-Maia Filho

**Affiliations:** 1 Universidade CEUMA, Post-Graduation Department, São Luis, MA, Brazil

## Abstract

**Background:**

New protocols are constantly being tested in the search for complete disinfection of root canals without the undesirable effects of sodium hypochlorite. This study evaluated the antimicrobial effect of ozonated water and photodynamic therapy (PDT) with sonic activation in root canals infected with *Enterococcus faecalis*.

**Material and Methods:**

Seventy single-rooted canals of human teeth were prepared and contaminated with *E. faecalis* for 21 days. The teeth were divided into six experimental groups (n=10): ozonized water without (O + S-) and with sonic activation (O + S +); PDT without (PDT + S-) and with sonic activation (PDT + S +); PDT + ozonized water without (PDT + O + S-) and with sonic activation (PDT + O + S +); and two control groups, one positive (n = 5) and one negative (n = 5). Microbial collections were performed before and shortly after treatment, counted in the log of colony-forming units and tested for significant difference between these counts, if any (Student’s t-test). The ANOVA two-way test was applied to evaluate whether the Treatment factor (Ozone, PDT and Ozone + PDT) and the Sonic factor (With and without sonic activation) had any effect on microbial reduction.

**Results:**

In all protocols, there was a significant microbial reduction (*p*=0.025), whereas in the groups in which sonic activation was used, the microbial reduction was significantly greater (*p*=0.001).

**Conclusions:**

The treatments significantly reduced the number of microorganisms in the root canals. Sonic activation helped to increase the microbial reduction in infected root canals.

** Key words:**Endodontics, ozone, photodynamic therapy, enterococcus faecalis.

## Introduction

The main objective of endodontic treatment is to eradicate or substantially reduce the microbial load in the root canal (1). However, the presence of bacterial biofilm inside and outside the root canals is an important concern in endodontics and is directly related to the index of success in endodontic treatments (2). In this sense, *Enterococcus faecalis* is present in up to 70% of endodontic failures due to its ability to form biofilms, as well as its high resistance to endodontic irrigators (3).

Endodontic irrigators must exhibit powerful antimicrobial activity, provide sufficient disinfection of the root canal, dissolve remnants of organic tissues, and have no cytotoxic effect on periradicular tissues (4). Sodium hypochlorite (NaOCl) is the irrigant of choice due to its great antibacterial effects and tissue dissolution (5), but it is toxic at high concentrations (6), in addition to weakening dentin, reducing its flexural strength and resilience, and making teeth more susceptible to deformation and possibly fractures (7).

Additionally, it must be considered that instrumentation alone does not seem to be efficient in removing organic and inorganic remnants from the root canals. Thus, during preparation of the root canal, cleaning could be complemented by agitation of irrigation solution, with techniques such as passive ultrasonic irrigation and sonic irrigation (8). Moreover, sonic irrigation reduces the risk of irrigation extrusion and damage to the root canal wall since it uses a plastic tip to agitade the irrigation solution (9).

Photodynamic therapy (PDT) has become an alternative method to enhance the disinfection of root canals (10). PDT uses a nontoxic photosensitizing agent (FS), sensitive to a specific wavelength of light, which produces cytotoxic reactive oxygen to some species of microorganisms and promotes efficient microbial reduction (11). However, for the PDT to be effective it is essential that the light source interacts with the FS agent; therefore, the choice of the light source depends especially on the FS that is used.

In this sense, erythrosine is an FS agent widely used due to its low toxicity and effectiveness in inhibiting the activity of oral gram-positive and gram-negative microorganisms and is approved by the Food and Drug Association for use in food products and in dentistry. In addition, it has an absorption peak very close to the emission of light sources used in dental offices (LED or halogen light sources with wavelengths between 500 and 550 nm), allowing the inclusion of this practice in the dental context (12).

On the other hand, ozone (O3) is a powerful oxidizing agent (13) used in the water industry to eliminate bacteria. Ozone therapy is based on the assumption that ozone dissociates quickly in water and releases a reactive form of oxygen that can oxidize cells, showing antimicrobial efficacy without inducing resistance to irrigants (14). In addition, one of the properties of aqueous ozone is its non-toxicity to oral cells *in vitro* (15).

Information concerning the antimicrobial effect of PDT (16) is rather limited and, at times, conflicting, as is the use of ozone for treating root canals (17). Accordingly, the aim of the present study was to evaluate the antimicrobial action of ozonated water and photodynamic therapy with erythrosine in root canals infected with *E. faecalis* using sonic activation.

## Material and Methods

-Selection and standardization of specimens 

After approval by the Research Ethics Committee (CEP) of the CEUMA University (UNICEUMA), under Opinion No. 2.868.372, 70 single-rooted teeth extracted from humans (incisors and canines) were obtained through the donation of teeth.

All had straight channels, without endodontic filling, fracture, or internal or external resorption, and were sterilized in an autoclave at 121°C for 20 minutes (min). Their crowns were sectioned using an Isomet 1000 precision cutting machine (Buehler Ltd., Lake Bluff, IL, USA), establishing a standardized root length of 13 millimeters (mm).

In order to confirm the apical patency, the root canal was explored with a K # 10 file (Dentsply-Sirona, Ballaigues, Switzerland) up to the working length (WL), which was standardized at 12 mm for all teeth. Root canal instrumentation was performed with the Reciproc® system (VDW, Munich, Germany) with an R40 instrument. For irrigation, 2 milliliters (mL) of 2.5% sodium hypochlorite was used, and aspiration was performed with 29-gauge 25 mm NaviTip® tips (Ultradent, South Jordan, UT, USA) and an aspiration-irrigation kit (Ultradent, South Jordan, UT, USA). After instrumentation, the root canals were irrigated with 1 mL of 17% EDTA (Biodinâmica, Ibiporã, PR, Brazil), which remained for 3 min after being agitated with the aid of a manual file, and at the end of the preparation, the canal was irrigated again with 3 mL of saline solution.

Subsequently, all teeth were kept in sterile saline with daily changes for 5 days to remove residual sodium hypochlorite. After this period, the teeth were apically sealed with Opallis® photopolymerizable composite resin (FGM Produtos Odontológicas Ltda, Joinville, SC, Brazil) and externally waterproofed with two layers of nail polish (Procosa Produtos de Beleza Ltda., São Paulo/SP, Brazil). The teeth were again sterilized in an autoclave at 121ºC for 20 min.

-*E. faecalis* culture

Microbiological procedures were performed in an aseptic environment inside the laminar flow chamber (BSTec, Brazil). Pure cultures of *E. faecalis* (ATCC 29212) were reactivated in Tryptic Soy Broth - TSb (Difco, Detroit, MI, USA) for 48 hours (h). The bacteria were inoculated into Tryptic Soy Agar - TSa plates (Difco, Detroit, MI, USA) and incubated in microaerophilia at 37°C for 24 h, forming a bacterial suspension prepared in sterile saline solution, with a concentration equivalent to 108 bacteria/mL. The optical density of the suspension was adjusted using the Macfarland Scale 0.5.

-Biofilm formation of *E. faecalis*

Biofilm formation was carried out by application of the pure bacterial suspension of *E. faecalis* in TSb culture medium until the root canal was completely filled using a 0.5 mL disposable syringe (Becton Dickinson, Campinas, SP, Brazil). Every 48 h for 21 days, the culture medium was reinoculated in the specimens to form the biofilm. At each replacement of the culture medium, a sterile cotton ball was placed at the entrance to the root canal. All specimens were kept closed in a microaerophilic environment and in an oven at 37ºC.

-Distribution of experimental groups

The specimens were randomly divided into groups according to the variables of interest: treatment group (Ozone, PDT, Ozone + PDT) and the presence or absence of sonic activation. The division of experimental groups is shown in Table 1.

**Table 1:**
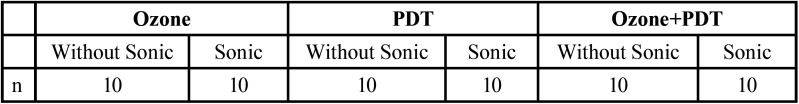
Division of experimental groups.

-Control groups

For the positive control (n = 5), canals were irrigated with 5 mL 2.5% NaOCl over 1 minute, while for the negative control (n = 5), the specimens received only contamination by the suspension of *E. faecalis*.

Methodology for LED application, pre-irradiation time and photosensitizing agent

To perform the PDT, the same operator used a high-intensity LED (Valo, Ultradent, UT, USA) with a wavelength of 460 nanometers (nm), adjusted to the Xtra Power (3200 mW/cm2), positioned and activated at the entrance of the canal, cervical region of the tooth, and 20 applications were made followed by a 3 seconds (s) pause each time, totaling 1 min.

Erythrosine B (Sigma Aldrich, St Louis, MO, USA), at a concentration of 200 micromolars (µM), was used as a photosensitizing agent, with a pre-irradiation time of 5 min.

-Ozonated water production

An ozone generator (Medplus MX, Philozon Eletroterapia, SC, Brazil) was used. For the production of ozonized water, 250 mL of autoclaved distilled water was used in a system with a glass tube coupled to the ozone generator, in which the gas was bubbled into the water, thus producing 40 parts per million (ppm) of ozonized water.

-Sonic activation

The same operator used EndoActivator (Dentsply-Sirona, Ballaigues, Switzerland), in high mode, with a 25/04 tip (medium). After the root canal was filled with aqueous ozone or erythrosine, it was agitated with sonic vibration with back-and-forth movements up to 2 mm from the working length, and two 30 s applications were performed, totaling 60 s of application.

-Microbiological collections and analysis

In all groups, two collections were made for microbiological evaluation. The first was performed before treatment and the second immediately after treatment. Both experiments were performed using two #40 sterile absorbent paper cones (Dentsply-Maillefer, Ballaigues, Switzerland) after the root canals were previously filled with sterile saline. The cones were transferred to polypropylene Eppendorf tubes (Cral, São Paulo, SP, Brazil) with 1.5 mL of sterile saline solution and agitated (Vortex AP 56, Phoenix, USA) for 1 min, with subsequent serial dilutions being performed and sown in petri dishes with TSa medium in triplicate. The plates were incubated in microaerophilia at 37ºC for 48 h. The results were obtained by counting colony-forming units (CFU) per mL and adding 1 ([CFU/mL]+1), and were then transformed into logarithms.

-Statistical analysis

After verifying that the data had a normal distribution (Shapiro Wilk, *p*> 0.05), we used Student’s t-test to test whether there was a significant difference within the groups between the initial microorganism counts and immediately after treatment.

Microbial reduction values were calculated by decreasing the initial values from the final microbial count values, and the ANOVA two-way test with Tukey’s post hoc test was used to assess whether there was a significant difference in microbial reduction between the experimental groups (Ozone, PDT, and association Ozone + PDT) and to determine whether sonic activation significantly altered the reduction of microorganisms.

The statistical program used was SPSS 26.0 (IBM, Armonk, NY, USA). The level of significance was set at 5%.

## Results

Table 2 shows the mean values (standard deviation) of CFU/mL log for the groups obtained at the two evaluation times. There was a significant difference between the initial and final values of the number of microorganisms in the root canals in all experimental groups.

**Table 2:**
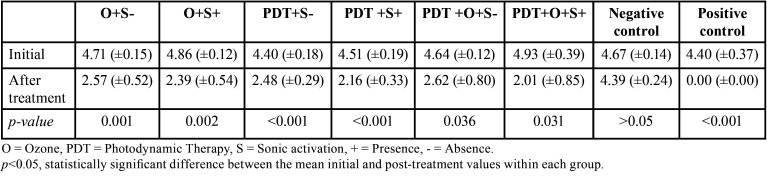
Mean values (standard deviation) of log ([CFU/mL]+1), in the different experimental groups at the two evaluation times.

**Figure 1:**
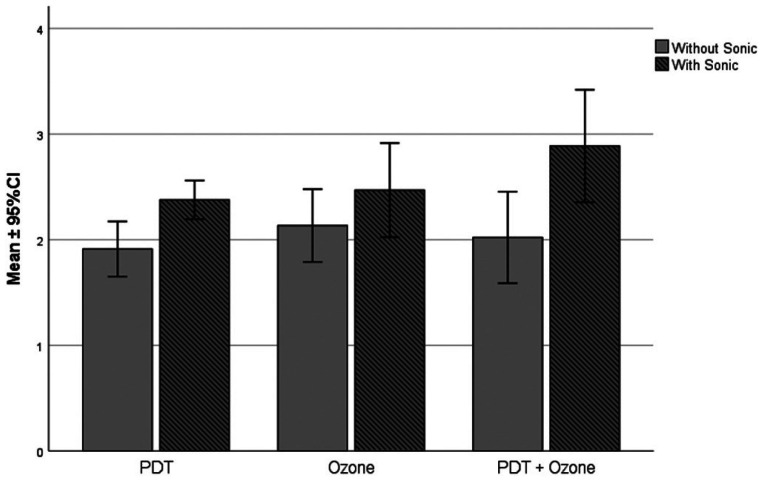
Behavior of the mean values (95% confidence interval) of microbial reduction expressed in log CFU/mL according to the experimental groups evaluated and the use of sonic activation.

Figure 1 represents a bar graph showing the average values of microbial reduction, in log CFU/mL, together with their respective 95% confidence intervals, according to the experimental group evaluated and the use of sonic activation.

The result of ANOVA two-way test showed that there was a difference between the treatment with Ozone, PDT and Ozone+PDT (*p*=0.025), and the sonic activation caused the average values of microbial reduction to increase significantly (*p*=0.001). However, the interaction (treatment and sonic activation) was not significant (*p*=0.220); that is, the factors acted independently on the reduction of microorganisms.

In the two-by-two comparison between the experimental treament with Ozone, PDT and Ozone+PDT, it was found that while the Ozone + PDT group was significantly different from the PDT group (*p*<0.05), there was no difference for the Ozone group (*p*>0.05).

## Discussion

The present study evaluated the antimicrobial action of ozonated water and photodynamic therapy with erythrosine in root canals infected with *E. faecalis* using sonic activation. All experimental groups achieved a significant microbial reduction, but there was complete elimination in none of the groups. In relation to the control groups, the reduction was not significant in the negative group. However, in the positive control group, which used sodium hypochlorite, there was a total bacterial elimination. This result indicates that the methodology was correctly used and that the results achieved were only due to the variables tested. Moreover, this outcome agrees with that of Muller *et al*. (18) where they found that only NaOCl was capable of completely eliminating biofilm from sundry bacteria.

Although infection of the root canal is often characterized by a biofilm of multiple species, a biofilm from *E. faecalis* was used in this study, mainly because it is the main pathogen linked to the failure of endodontic treatment, in addition to its ability to penetrate the dentinal tubules, colonize the root canal system, and form biofilms (19).

The period of 14 or 21 days is sufficient time for the formation of *E. faecalis* biofilm on the dentin substrate (20). In this study, a culture period of 21 days was adopted, allowing bacterial growth and ensuring that the decontamination protocols could be tested.

In this study, two complementary therapies were used in an attempt to find an effective protocol to combat endodontic infections caused by *E. faecalis*. PDT was performed with the application of erythrosine (200 µM) as FS, sensitized with high-intensity LED with a wavelength between 500 and 550 nm, the highest absorption range for erythrosine (21). The choice of FS was due to the promising results reported in other studies (12,22).

Fracalossi *et al*. (23) evaluated the singlet oxygen generation of erythrosine solutions illuminated with halogen light and found that the minimum inhibitory concentration of erythrosine varied between 0.312 and 0.156 mg/mL. In view of this observation, 200 mM (0.167 mg/mL) was used in this study.

The LED was used at its maximum power (3200 mW/cm²) for 60 s. Although PDT has shown significant results for bacterial reduction of the root canal, it has failed to reset microbial growth. In contrast, Borba *et al*. (22) achieved a 100% reduction in *E. faecalis* using a high-power LED and erythrosine. However, they used an exposure time of 120 s and used *E. faecalis* in planktonic form, which is easier to eliminate.

Ozone has been suggested for use in endodontic treatment due to its high antimicrobial action (24) and because it presents a significant decrease in cytotoxicity to oral mucosa cells, compared to other irrigating solutions like NaOCl (2.25%) and chlorhexidine (2%) (15). Although bacterial reduction in our study reached significant levels with ozone, it was not able to produce results similar to those of Hubbezoglu *et al*. (25), who used a concentration of 16 ppm of aqueous ozone solution activated with ultrasonic irrigation technique, which achieved a microbial reduction of 100%. The difference between the results may be due to the application time used. While Hubbezoglu *et al*. (25) used an application time of 180 s, in this study, 60 s was used.

When sonic activation was used, the groups achieved higher mean values of microbial reduction (Fig. 1). In addition, Eggmann *et al*. (26) found a significant impact of sonic irrigation on microbial reduction in channels infected with *E. faecalis*, **Candida* albicans*, *Streptococcus gordonii*, and *Actinomyces oris*. However, Plotino *et al*. (27) evaluated the effectiveness of the activation of irrigants in a sonic and ultrasonic manner and concluded that the ultrasonic system was more effective in removing the remains of pulp tissue and dentin, however, they deformed the root canal.

Although several studies have shown the effectiveness of using PDT (28,29) and ozone therapy (30), none have evaluated the effect of the association between these techniques. The present study showed that the association between PDT and ozone therapy may be an alternative to endodontic treatment; however, it is recommended that other parameters in relation to the property of the light used, exposure time, photosensitizers, and ozone concentration, including the use of other microbial strains, be tested to obtain more satisfactory results, both from PDT with erythrosine and ozonated water, and thus seek an effective and nontoxic antimicrobial agent.

## Conclusions

All treatments tested were able to significantly reduce the number of microorganisms in the root canals, especially when used with sonic activation. The association of ozone + photodynamic therapy with 200 µM erythrosine reached the highest mean value of microbial reduction.
